# Changes in perceptions of the alcohol environment among participants in a Photovoice project conducted in two districts with different socio-economic status

**DOI:** 10.1371/journal.pone.0254978

**Published:** 2021-08-06

**Authors:** Irene Molina-de la Fuente, Andrea Pastor, Paloma Conde, María Sandín Vázquez, Carmen Ramos, Marina Bosque-Prous, Manuel Franco, Xisca Sureda

**Affiliations:** 1 Public Health and Epidemiology Research Group, School of Medicine, Universidad de Alcalá, Alcalá de Henares, Madrid, Spain; 2 Malaria and Neglected diseases Laboratory, National Centre of Tropical Medicine, Institute of Health Carlos III, Madrid, Spain; 3 Department of Biomedicine and Biotechnology, University of Alcalá, Alcalá de Henares, Madrid, Spain; 4 Public Health Institute of Madrid, Madrid City Council, Madrid, Spain; 5 Faculty of Health Sciences, Universitat Oberta de Catalunya, Barcelona, Spain; 6 Department of Epidemiology & Biostatistics, Graduate School of Public Health & Health Policy, City University of New York, New York, United States of America; 7 Department of Epidemiology, Johns Hopkins Bloomberg School of Public Health. Baltimore, Maryland, United States of America; 8 Tobacco Control Research Group, Institut d’Investigació Biomèdica de Bellvitge-IDIBELL, l’Hospitalet de Llobregat, Barcelona, Spain; 9 Consortium for Biomedical Research in Respirarory Diseases (CIBER en Enfermedades Respiratorias, CIBERES), Madrid, Spain; City University of New York Graduate School of Public Health and Health Policy, UNITED STATES

## Abstract

Perceptions of the alcohol environment may influence alcohol consumption patterns. The purpose of this study was to describe changes in perceptions of the urban alcohol environment as experienced by residents of two districts with different socio-economic status after taking part in a Photovoice study. The study was conducted in Madrid, Spain, in a district with a high socio-economic status (HSES) and another district with a low socio-economic status (LSES). A Photovoice project was conducted with 26 participants divided into four groups based on sex and district. Groups met over five sessions in which they discussed photographs taken by the participants themselves on the subject of alcohol in their neighbourhood. A qualitative, descriptive and thematic analysis of participants’ discourses was performed to explore changes in their perceptions of the alcohol environment over the project sessions. Changes in perceptions of the alcohol environment were observed in all groups over the project. The process of change varied by districts’ socio-economic characteristics and gender. Greater changes in perceptions of the alcohol environment were observed in HSES, especially among women, as the participants had a much more positive initial view of their alcohol environment. In LSES, participants showed a more critical perception of the alcohol environment from the beginning of the study, and this broadened and intensified over the course of the sessions. Changes in perceptions also varied by thematic categories, including some categories that were discussed from the start (e.g. socialising and alcohol consumption) and categories that only emerged in later sessions (e.g. alcohol advertising). Involvement in a Photovoice project has favoured a shift in the participant’s perceptions of their alcohol environment towards more critical positions, widening their scope of perceived elements and raising their awareness of specific problems, such as alcohol advertising and social role of alcohol consumption in relation to alcohol exposure.

## Introduction

Alcohol consumption has been identified as a major risk factor for global health, causing approximately three million deaths every year [1]. Alcohol’s perceived risk and social image may shape drinking patterns [2, 3].

Normalised alcohol consumption and low risk perception are associated with higher levels of heavy drinking [4]. This association is particularly problematic among young people, where it has been linked to binge drinking [5]. In Spain, alcohol consumption enjoys a high degree of social acceptance, as evidenced by people’s tendency to underestimate their own intake [6, 7]. Social acceptance of consumption may be influenced by the urban alcohol environment, as well as by the social and cultural context [8, 9]. Key characteristics of the urban alcohol environment include alcohol accessibility and availability [10, 11], as well as advertising in public spaces [12, 13].

Most studies that have sought to determine social perceptions of alcohol and drivers of consumption have been based on surveys [14, 15] or personal interviews [16]. Other studies have assessed the effect of preventive actions on drinking patterns [17, 18]. This study reports on how involvement in a participatory action research study affects participants’ perceptions of their alcohol environment.

The Photovoice methodology makes possible to use photography to capture people’s perceptions and document the features they think that are related to a particular community issue [19, 20]. In parallel, the participant population undergo a process of reflection and learning [21]. This technique also favours a stronger critical awareness in the participants, as well as a stronger commitment to achieve common objectives aimed at improving their environment, as a result of their empowerment [22]. The Photovoice methodology has already been widely used in the past to characterise residents’ perceptions on their urban environments [23–26].

The purpose of this study was to describe changes in perceptions of the urban alcohol environment as experienced by Photovoice participants over the five project sessions. This study was conducted in two districts of Madrid with different socio-economic status. The results of these changes have been assessed by means of qualitative analysis of the participants’ discourses, by sex, district and topics discussed.

## Methodology

### Study design

This Photovoice study of the urban alcohol environment was conducted in two districts of the city of Madrid with different socio-economic status in order to assess if perceptions of the residents’ alcohol environment changed depending on the socio-economic characteristics [26]. We selected two districts with a long history of local participation to facilitate the recruitment of the participants and the Photovoice process.

Chamberí, had a population density of 294 inhabitants/ha, of whom 10.4% were foreign-born. The rate of residents with low educational level (elementary studies or below) was 7.61% and the unemployment rate was 6.13%. In comparison, Villaverde had a population density of 72 inhabitants/ha, of which 18.0% were foreign-born. The rate of residents with low educational level was 27.95% and had an unemployment rate of 11.53%. Data were extracted from the Madrid Municipal Registry (https://www.madrid.es/portales/) updated to 2018. Chamberí is a high socio-economic status district (HSES) with tourist attractions and a high purchasing power while Villaverde is a low socio-economic status district (LSES) in the city outskirts.

We conducted this study in accordance with the Declaration of Helsinki, and received ethical approval by the Ethics Committee of the Universidad de Alcala [CEI/HU/2017/09].

### Participants

The participants were recruited through purposive sampling [27] of volunteers. The inclusion requirements were as follows: 1) has lived in the district for more than one year; 2) aged between 40 and 75; 3) able to communicate in Spanish; 4) able to use a camera without any problems; 5) agrees to attend a minimum of 5 sessions over 5 consecutive weeks.

The age range was limited to people between 40 and 75 since this research is a sub-study of the Heart Healthy Hoods project (hhhproject.eu), aiming to understand how the urban environment relates to cardiovascular health in adult population of these ages. Finally, the age range was 39–78 due to difficulties for the recruitment.

### Photovoice methodology

Four Photovoice groups were formed based on residence district and sex. Each group met in a minimum of 5 sessions of approximately two hours over consecutive weeks. These were held in 2017 in HSES, in a Social Service Centre, and in 2018 in LSES, in the Municipal Community Health Centre. Participants were recruited by research team and the Public Health Technicians using a purposive sampling strategy, based on leaflets distribution and briefings. Two team researchers acted as facilitators in all the sessions, speaking as little as possible to give the participants the leading role.

The methodology was flexible enough to structure the sessions according to the groups’ needs. The sessions were audio recorded and transcribed for subsequent analysis.

In the first session, the research team introduced the project and the Photovoice methodology and encouraged them to photograph everything related to alcohol in their neighbourhood. In the same session, all the participants received photography training and signed an informed consent to take part in the study. In sessions 2 to 4, the participants presented, using an adapted version of the SHOWED questionnaire [28], the five most relevant photographs they had taken during the previous week, and these were then discussed. The questionnaire included the following three questions: 1) What does the photograph show?; 2) What is the story behind it?; 3) What is the connection with alcohol?

In sessions 3 and 4, the participants grouped the photographs into topics. In session 5 and extra sessions when needed, these topics were classified into final, more general thematic categories, and each one was assigned a title and a representative photograph.

Lastly, a joint meeting was held in each district bringing both groups (men and women) together. This meeting had the following goals: 1) to meet and to share experiences; and 2) to share the results of the final thematic categories to produce a common mind map for each district.

Finally, Photovoice participants developed policy recommendations in two extra sessions as a form of community-based solutions to improve their alcohol environment.

### Data analysis

A qualitative, descriptive and thematic analysis of the participants’ discourses was performed [29]. All sessions were transcribed, and the discourses were coded using a deductive approach based on the final thematic categories resulting from the Photovoice process, as established by the research team [26]. Subsequently, changes in the discourses on each thematic category were identified, taking into account the participants’ district of residence and applying gender perspective. Researcher triangulation was used to ensure a high standard in the quality and validity of the analysis [30]. To enrich the report and increase the reliability of the results, we have included original transcribed sentences and photos of the participants that accurately represent the group discussions. These quotes were translated from Spanish to English.

## Results

### Description of the participants and the sessions

A total of 26 people (7 women and 6 men per district) between the ages of 39 and 78 took part in the project. Two people were accepted who did not meet the age requirements, in order to have an optimal number of participants. Of the 12 HSES participants for which educational and income level information was available, 10 had studied at university and 7 had monthly household incomes of more than 2,200 euros. In LSES, 5 out of 13 participants had vocational training as their highest educational level, and 7 out of 13 had a monthly family income of less than 1,700 euros. All the participants were born in Spain.

We adapted the dates of the sessions to ensure that most of the participants could attend (at least 5 participants per session). Attendance was almost fully in all sessions, and none of the participant missed more than 2 sessions. Only one participant dropped out of the study after the 3rd session due to work reasons (one man from HSES District).

### Changes in the participants’ perceptions over the Photovoice study

Perceived alcohol exposure changed over the course of the Photovoice study. Moreover, their discussions on alcohol urban environment became increasingly critical.

The shift in perceptions is reflected in the differences we found in their discourses over the course of the sessions. Firstly, we observed changes in discourses on topics that had been perceived from the outset. Secondly, we observed new topics that had not been discussed in the initial sessions. Moreover, the final sessions reflected a much more critical view of alcohol in both districts.

In the joint sessions, the participants mentioned that, since they had been involved in the study, they had started to identify more elements of the environment as being linked to alcohol: *“I pay more attention now*, *and I like it because lots of things never used to strike me*, *and now they do*. *This has awakened my vision in that regard*.*”* (Woman, HSES, joint session). During the course of their involvement in this study, they also reported having gradually become aware of how alcohol is present in their districts and in their daily lives: “*It struck me how alcohol can sit with all kinds of things*. *I wasn’t aware of how alcohol finds its way into any place you can think of*.” (Man, HSES, joint session). The participants also acknowledged that the group discussions had been key in broadening their perceptions: “*Looking at the same picture*, *when we discussed it as a group*, *points were made that you’d never given any thought to*. *So you start seeing new things*.” (Woman, HSES, joint session). They particularly valued the fact that the study was performed by the residents themselves, whose participation could potentially encourage the public’s involvement in the well-being of their environment: “*It needs to be locals who take part*, *because they’re the people who know the area*.” (Woman, LSES, joint session).

These two phenomena, the change in perception and the positive assessment of their participation, occurred equally in both districts.

### Changes in the participants’ perceptions by district and gender

Changes in perceptions varied with the socio-economic status of the district of residence and gender. They were also strongly influenced by each group’s initial perceptions of the alcohol environment. In both districts, the women’s group underwent a more marked transformation than the men’s group.

Major differences were observed in the HSES women group’s perceptions of the alcohol environment over the course of the sessions. In the initial sessions they showed very little awareness of their exposure to alcohol and a positive perception of everything related to alcohol in their district. Many new topics emerged as the sessions progressed, and they took on a more critical stance, focusing on negative aspects of their alcohol environment. For example, at first, participants identified new forms of drinking in their district relating to consumption of gourmet alcohol targeted at higher social classes: *“These wine tastings are a great idea*. *You see them everywhere*, *it’s really trendy now*.*”* (Woman, HSES, session 3). They highlighted how alcohol consumption can be combined with cultural and educational activities, which makes drinking a more enriching experience. For example, they made reference to bookshops offering reading sessions and language exchanges with alcoholic drinks: *“This bookshop-bar is a place where you become educated and drink too*. *Knowledge and alcohol*, *both are exchanged*, *you see*?*”* (Woman, HSES, session 4). All this exposure was perceived as positive, exclusive, representative of their district, and linked to socialising around alcohol. But, as the sessions progressed, they perceived that these new forms of consumption had introduced alcohol into certain spaces and activities that had previously been drink-free and were potentially encouraging its acceptance and normalisation: “*A small traditional grocer’s in Vallehermoso market has become a wine-tasting place […] It’s shocking*, *it looks like there’s more alcohol in the market than fish nowadays*! *Food markets are being turned into places for drinking*.*” (Woman*, *HSES*, *session 5)*.

Conversely, the HSES men’s group displayed the lowest degree of change in perceptions and in acquiring a critical discourse on the alcohol environment, based on a comparison of the topics discussed over the course of the sessions. From the outset, they did not see any problems in relation to their district’s alcohol environment and pointed out alcohol’s socialising role as a benefit. The topics that emerged in the initial sessions continued to be the focus of discussion in subsequent sessions, with hardly any changes in discourses throughout the process. These participants did discuss, nonetheless, some aspects of alcohol which they had not thought about before taking part in the project. In the final sessions, they identified certain aspects of alcohol as problematic. For example, whereas at first they did not pay attention to how bar and restaurant terraces in their district “invade public spaces”, this became a subject of discussion and concern in later sessions: *"All terraces are a nuisance to residents [*…*] They take up more than half the space on the pavement*. *They’re the ones people complain about*, *because they can’t get past when they’re pushing trolleys*, *for instance [*…*] They’re in the middle of the street*, *people chatter*, *it gets noisy*, *and it’s just an inconvenience*, *particularly for the local residents*.*”* (Man, HSES, session 4).

Change followed a different pattern in LSES where, from the initial sessions, both groups showed a much greater degree of awareness in their collective perception of the alcohol environment in their district and potential problems related to it. The participants were less accepting of exposure to alcohol and more concerned about its strong presence in their district. Their perception did not change as markedly as in the HSES women’s group, but it did broaden to take in new topics that they had not perceived in the initial sessions. One example of this was the subject of alcoholism, which since the start of the study had been linked to negative social and economic consequences both at the individual and the collective level: *"He looks in really bad shape*. *I reckon it’s because of drinking*. *This bar in particular attracts quite a large crowd*. *I see them every day*.*”* (Man, LSES, session 3). Over the course of the Photovoice process, this subject expanded to include related topics such as "Alcoholism and other addictions" and "Rehabilitation". The first of these focused on the link between alcohol consumption and gambling addiction, specifically in betting establishments. The second, on the involvement of the district community in the rehabilitation of alcoholics.

### Changes in the participants’ perceptions by final alcohol thematic categories resulting from the Photovoice process

In addition to district- and gender-based variations, we observed differences in the participants’ discourses by topics related to the alcohol environment. Table 1 contains verbatim transcriptions of the participants’ statements as examples of the changes in their discourses on some of the thematic categories discussed. We selected phrases said by different participants that we consider representative of their photovoice group. Topics that were not obvious at the start emerged in later sessions, and discourses on some topic that emerge from the first sessions evolved over the course of the sessions and over the entire study process.

**Table 1:** Changes in the participants’ perceptions by topics.

Topic	District	Gender	Initial sessions (Sessions 2–3)	Final sessions (Sessions 4–5 and extra sessions)
**Advertising**	High Income District	Women	“A small traditional grocer’s in Vallehermoso market has basically become a wine-tasting place. It’s beautifully designed, just looking at the labels on the wine bottles draws your attention.”	“There was so much advertising I made a collage about it. The casks that Mahou beer put up, the sunshades and chairs with the Mahou logo on them, and the sign with the day’s set meals and a beer on it too … So beer is advertised everywhere, in the street […] You’re being pushed to drink." (Fig 1A)
		Men	Not discussed	“Maybe we’ve just internalised it or learnt to live with it. But given the major role alcohol has in our society, I expected to find a lot [of advertising] […] Instead, I basically saw advertising on awnings, tables and napkin holders, not massive posters as I expected.” (Fig 1B)
	Low Income District	Women	“It struck me because it’s true that you come in and it’s as if you’re enticed to order a beer. It’s the power of advertising and marketing with alcohol, isn’t it?”	“It’s an establishment with a sign saying ‘Groceries’. But the only thing in the shop window is alcoholic drinks […] I’ve given it the title ‘Groceries?’ because actually it just encourages drinking.” (Fig 1C)
		Men	“You wouldn’t think so, but we have a lot of subliminal messages walking round the streets. I saw a lot of them about alcohol […] Beer brands use marketing and advertising to try to build associations, create customers, make them loyal …”	"It caught my attention because the slogan is ‘Celebrate what we are’. It’s for a [Spanish] DYC whisky ad showing a football pitch where alcohol is everywhere. So my question is: What exactly are we celebrating? Or what exactly are we? It’s clearly encouraging people to drink, and it also sends out that message …” (Fig 1D)
**Socialising**	High Income District	Women	"To celebrate a friendship, in a farewell get-together, there’s always, or nearly always, food and above all drink. It’s tied to our local culture, meeting for food and drinks and raising glasses. And toasting with water is bad luck." (Fig 2A)	"I’ve realised that we don’t know how to have fun without alcohol […] and that alcohol seems more harmless if it’s something you do to socialise."
		Men	"There’s a very lively social life because there are lots of venues of all kinds. Concert halls, places for young people, for older people, for dancing … There’s a bit of everything.” (Fig 2B)	“Alcohol is always linked to socialising. If there’s no alcohol, there are no get-togethers, no socialising, nothing.”
	Low Income District	Women	"We tend to be social drinkers and when we’re away from home and you meet friends for a chat and so on, you usually have a beer. It happens all the time. I call it being a social drinker, because real alcoholics drink at home *and* outside.”	"I believe alcohol used in the right measure, avoiding abuse, can help socialising. Or perhaps it is so much a part of our customs and socialising habits that it is often essential, so to speak. Yes, you can just have a soft drink, but people usually drink a beer or a vermouth. It’s as if it goes with the environment.” (Fig 2C)
		Men	“We went out together and we went for a beer. It was a good choice to drink that beer together in the bar. I believe there are times when alcohol can be a good thing.”	“People say ‘We’ll have to get together for a beer some time’. It’s an excuse to get together, meet up and chat for a while. The bar is, so to speak, the place, see? The place where you go to drink and socialise.” (Fig 2D)
**Children’s exposure to alcohol**	High Income District	Women	“There’s a time of day in bar terraces which is children’s time. […] They’re there with the push-trolleys, the kids, the grandparents. They’re family get-togethers.” (Fig 3A)	“I think we do pass it on to them. It’s part of our life to have a drink, and if children are around it’s no big deal. It’s really normalised. […] Although this may change in a few years and it will be seen as a negative thing, since it is a bad example for kids, just as smoking.”
		Men	Not discussed	“All things are fine in the right measure. But if it’s so close, you can see it in front of you, you’re being bombarded, it gets to you. It might get to a child from this area more than it gets to me. I’ve lived in a more residential area.”
	Low Income District	Women	“They’re drinking alcohol at any age in the middle of the street.It’s their way of getting together, but they cause a nuisance. They encourage children to drink since they’re really small drinking in front of kids.*”*	“A children’s bottle with children’s decoration shouldn’t have the same format as a bottle of cider, for instance. Much less should it be kept next to alcoholic drinks. Children who drink those think they’re grown up because it’s the same shape as the drinks their parents have.”
		Men	“I, for instance, can’t see the positive side of this. A bar terrace full of kids, their parents drinking alcohol and the kids playing in the same place.”	“It’s part of their daily or weekend routine to say ‘Let’s go down to the bar for a beer’. You’re not thinking of the child. The kid comes with you and drinks the beer too. Not now, but when he or she grows up, because it’s a natural thing to do.” (Fig 3b)

**Fig 1:**
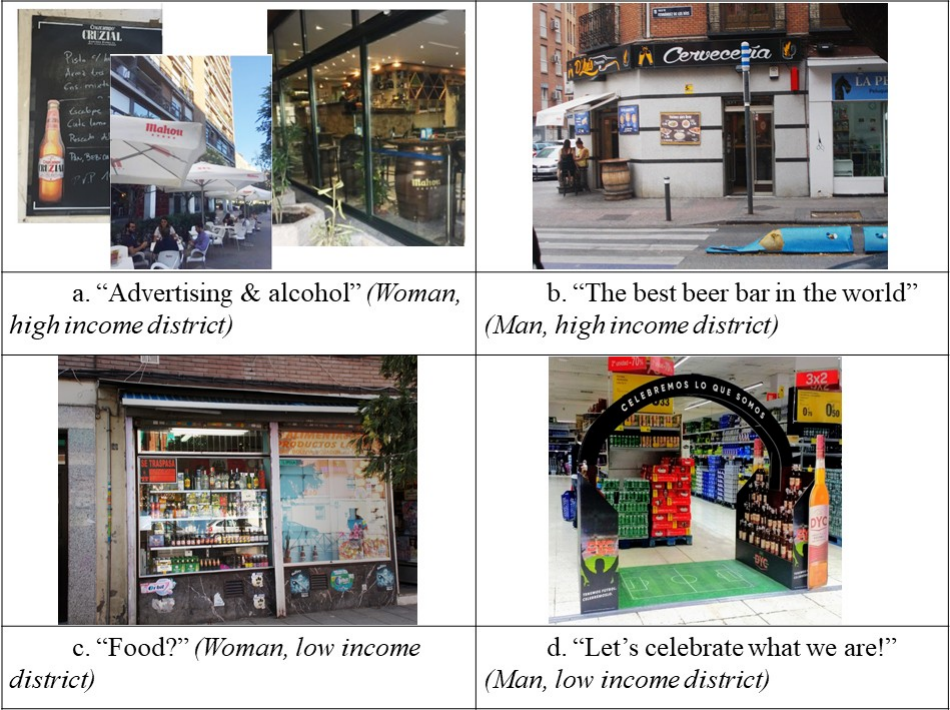
Photographs on alcohol advertising and promotion.

**Fig 2:**
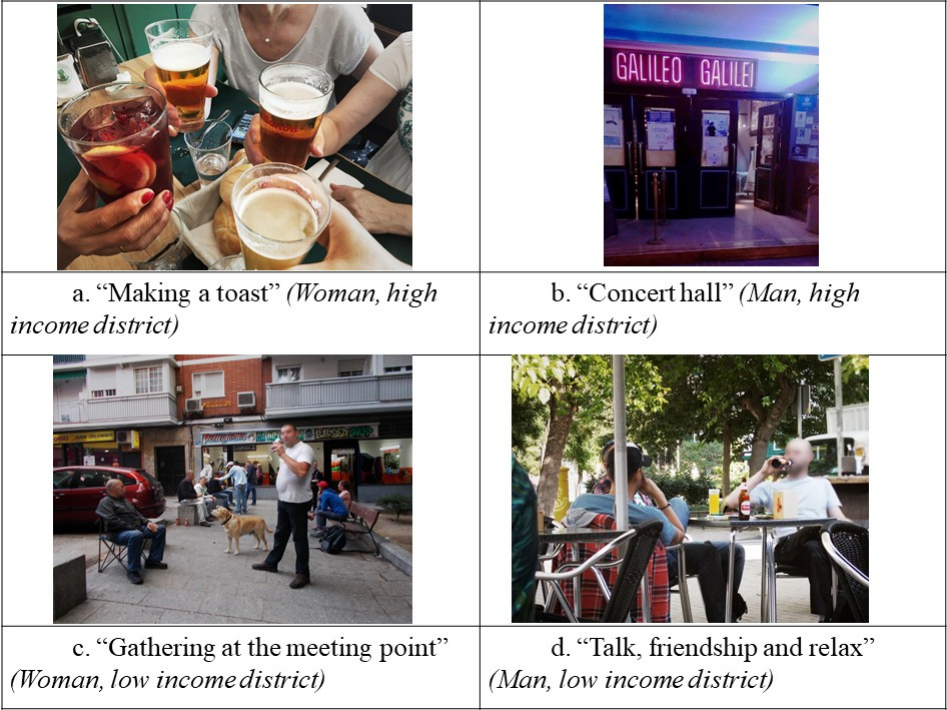
Photographs on the social role of alcohol.

**Fig 3:**
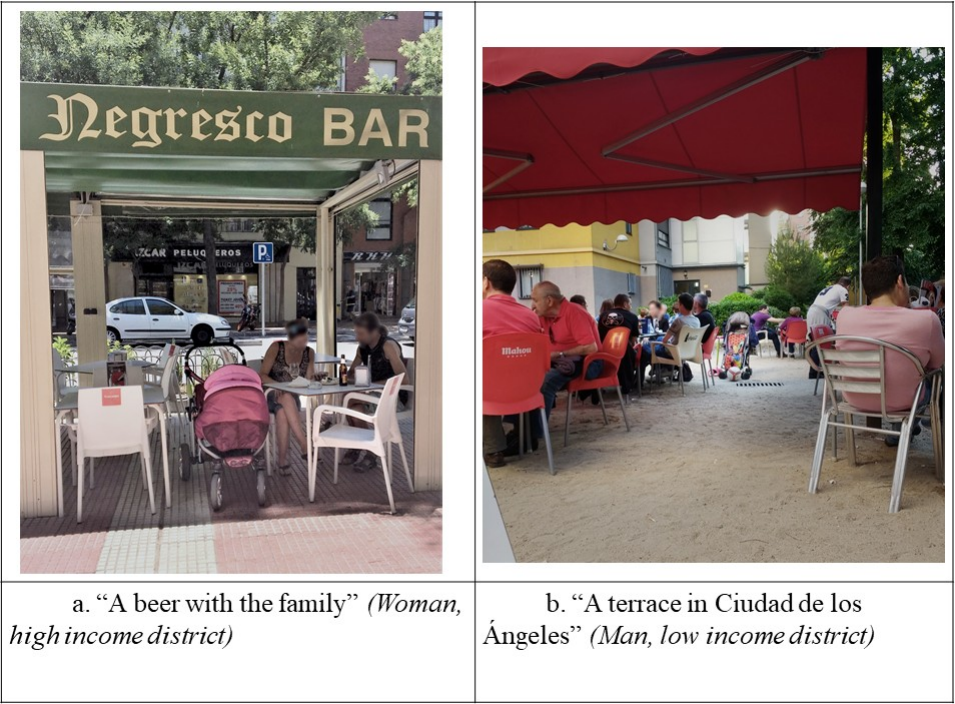
Photographs on children’s exposure to alcohol.

We observed that the topics most discussed in the sessions were those areas in which the participants’ perceptions changed more significantly. One of these topics were alcohol advertising. Here, perceptions changed gradually in all the groups. This aspect did not emerge in the initial sessions, or it was only present in the discourses of the youngest participants: *"The advertising on the sunshades*, *the casks and other bar furniture you see in the street*, *it all struck me [*…*] The thing is*, *I don’t think you make the connection at first sight*, *but it is all strictly bar furniture*, *isn’t it*?*”* (Woman, HSES, session 2). These interventions encouraged the rest of the group to take pictures of alcohol advertising. In the following sessions this topic were identified as a key source of alcohol exposure in their districts: *"I think*, *because it’s there all the time for you to see*, *it makes you crave for a beer*. *I’ve become very aware of that since I’ve been taking part in this*.*"* (Woman, HSES, session 5). Discourses on alcohol advertising became more critical in the final sessions: *“I see the power advertising has on individuals here*. *Say you’re thirsty and you feel like a glass of water*. *Then you see the advertising and all the rest*, *and it’s as if you need to go for a beer*.*”* (Woman, LSES, session 4).

Regarding the subject of the socialising role of alcohol, the shift in perceptions about it differed from change process in other topics. This topic emerged from the first session in all groups and was a recurring subject of discussion throughout the study. In the initial sessions, the participants only focused on the positive aspect of alcohol in social relationships: *"What I mean is that*, *wherever you go*, *social life and co-existence revolve around alcohol*. *It’s true alcohol brings people together*.” (Man, LSES, session 2). In later sessions, all the groups reflected on the idea that alcohol is implicit in, and almost necessary for, socialising: *“It seems to me that we have a society that unfortunately views sitting around a glass of beer as the means or the communication line needed for a social conversation*.*”* (Man, HSES, session 4). Even so, they still had a positive perception of what they referred to as “moderate” consumption for socialising, emphasising situations where alcohol is a means for celebration, group bonding or relaxation: “*It’s true sometimes having a beer and so on in good company can be positive*. *[*…*] I see all terraces as friends getting together*.*”* (Man, LSES, session 5).

Another topic on which the participants developed a more critical perception over the course of the sessions was the risks of children’s exposure to alcohol. This aspect emerged when participants from the different groups began to observe that drinking in front of children is commonplace. In the initial discussions, since this situation is part of everyday life, the participants did not consider it strongly negative: *" You’re out for ‘tapas’*, *you sit down at a terrace and order some beers with children around*. *Nobody’s going to think you’re setting a bad example for the kids*.*"* (Woman, HSES, session 4). However, in later sessions some discussions emerged about the negative implications that this highly normalised exposure to alcohol can have on children behaviours. For example, the participants discussed whether this behaviour can potentially influence children to view alcohol consumption as positive or whether it might encourage them to drink at a younger age as they try to mimic adults: *"A child in this area is more heavily bombarded with alcohol than one in a different district [*…*] It’s not normal for a park to be next to a bar terrace*, *but it’s become normalised [*…*] It’s convenient for parents but a bad example for children*. *Too much normalisation*.*"* (Man, HSES, session 5).

In later sessions, however, discussions emerged about the negative implications that this highly normalised exposure to alcohol can have for children.

## Discussion

The participants underwent a shift in their perceptions of the urban alcohol environment towards a broader, more critical outlook. This was facilitated by the conversations and discussions that were held in the sessions [21]. This participatory group process favoured increased sensitivity and awareness among the participants about the reality of their social context in relation to alcohol [22]. The groups also experienced changes in their opinions and feelings towards alcohol, both individually and collectively [31].

The Photovoice methodology gives visibility to community’s priorities and concerns about a particular issue [23]. Topics and situations that are most often discussed and photographed are precisely those where the most significant changes in people’s perceptions are observed, as they are higher up among their subjects of thought and reflection [32]. This is consistent with the results observed in our study. Moreover, participants increased their awareness about topics discussed through the Photovoice sessions.

Initial variations in perceptions of the risks linked to alcohol consumption also influence how these perceptions later evolve [33]. The women in HSES underwent stronger changes towards a heightened perception of risk in connection with alcohol consumption, as their starting position was one of greater normalisation and acceptance of drinking. This may be due to the tourist-oriented and social nature of the district, where the presence of alcohol is associated with culture, leisure activities and social status [34, 35]. Links between alcohol and social activities increase the acceptance of alcohol consumption. These changes in perceptions of the alcohol environment were not so obvious in the group of men in HSES, so it would be interesting to explore this issue further using a gender perspective.

In LSES, the participants showed a more critical perception of the alcohol environment from the outset. This may be because the negative impact of alcohol consumption is stronger in vulnerable areas [36]. Thus, having been more critical since the initial sessions, the shift in their perceptions of the alcohol environment was not as marked as in HSES. Still, they elaborated their views on the aspects that had been discussed from the start, and on other topics that emerged in later sessions. Previous Photovoice studies show that vulnerable populations differ from other populations in their perceptions of their environment and its problems [37, 38].

One aspect on which all groups changed their perceptions was alcohol advertising, albeit with variations depending on the district’s socio-economic status. This topic was either absent or very vague in the initial sessions but became a recurring issue in later sessions when the participants gained greater awareness about the subject of study. The fact that most participants did not perceive alcohol advertising at the beginning of the project may be due to its pervasiveness, which makes it an integral part of the urban environment (e.g. alcohol logos on bar tables, chairs and sunshades). Constant or repeated exposure to an element of the environment diminishes perception of that element and its potential risks, as explained by Social Cognitive Theory [39]. This indicates the need to strengthen policies regulating alcohol advertising and promotion in public spaces, including establishments where alcohol is sold and consumed. Increased presence of alcohol advertising in the urban environment has been shown to be linked with heightened alcohol consumption, especially among younger people [40, 41]. It would therefore be beneficial to implement health promotion actions to raise people’s critical awareness and sensitivity towards alcohol advertising.

Alcohol’s socialising role was the topic where the fewest changes in perception were observed over the course of the sessions. This may be due to alcohol consumption’s long-standing normalisation as positive and to its low-risk perception, being associated with moments of pleasure and relaxation [42]. The participants made many positive references to “moderate” alcohol consumption as a harmless drinking option. “Moderate” consumption is a term used by the alcoholic beverage industries to dampen risk perception and foster belief in "responsible, less harmful drinking" [43, 44], despite the evidence that no level of alcohol consumption is safe [45, 46]. It is also worth noting that viewing photographs showing everyday scenes in which alcohol was a common element prompted the residents to reflect on the negative effects of normalising its consumption. This reflection, influenced by the joint process experienced within each study group, is what facilitates individual, and collective, awareness about the scenes in the pictures [18]. The increased awareness could mean an in–depth change in their attitudes and habits in relation to alcohol.

Additionally, the participants showed a more critical perception of aspects that were new to them, issues they had not previously thought about, i.e. children’s exposure to alcohol environment. The fact that some elements have been identified for the first time in group discussions may have facilitated collective reflection and a more critical form of learning. Previous studies have observed Photovoice’s potential as a strategy for collective learning and critical reflection [47, 48].

The variations observed between women’s and men’s discourses could be explained, firstly, by their different perceptions of the alcohol and health environment. Women from both districts were more prompt to perceive the alcohol environment in terms of risk factors [49]. This may be linked to women’s greater exposure to certain social and health risks associated with alcohol [50, 51], or with the heightened risk of being assaulted in alcohol-related contexts [52]. Secondly, the higher degree of learning and perception development seen in women’s groups could be linked to a stronger predisposition to engage in Photovoice group discussions and reflection, partly favoured by their groups’ female-only make-up [53].

### Strengths and limitations of the study

The use of photography in this methodology facilitates discussion on the alcohol environment and resonates with emotions and experiences more universally, encouraging communication and reflection within the group [54, 55]. Moreover, this study included participants throughout the entire research process, ensuring that the results obtained are plausibly in line with their life realities and enabling the research team to discover the community’s problems and priorities without influencing the process [24]. A potential limitation of this study is that, although the participating population was suitable for the methodology [56], it may not be representative of the districts. Nonetheless, the purpose of this study was to elaborate on the topics presented for discussion, not to ensure representativeness. Another limitation is that all participants were over 40 years old, since this research is a sub-study of the Heart Healthy Hoods project (hhhproject.eu). Moreover, all of them were Spanish. It would be interesting in future studies to include participants from other ages and nationalities. In addition, it would be interesting to analyse if changes observed in the participants’ perceptions of their alcohol environment are maintained over time.

## Conclusion

Photovoice engagement favoured changes in the participants’ perceptions of their alcohol environment, widening their scope of perceived elements and intensifying their consideration as potentially relevant. This process resulted in more critical discourses on aspects of their alcohol environment that participants consider important, that means in-depth reflections and complex discourses about their alcohol environment and its negative impact on health. Results obtained in this study showed that this methodology increase participants critical awareness of their alcohol environment. Changes in perceptions were different by gender, with the groups of women undergoing greater changes in their perceptions of the alcohol environment.

Results also showed important differences based on the socio-economic characteristics of the two districts where the study was conducted. Groups in the district with a lower socio-economic status showed a more critical perception of the alcohol environment from the outset of the study. The groups in the district with a high socio-economic status had a more lenient perception of the alcohol environment, which evolved towards greater awareness of certain problems, such as bar terraces’ invasion of public spaces and alcohol advertising.

This study showed that participation in a Photovoice project encourages engaged residents to share their reflections and change their perceptions regarding their urban alcohol environment. The results obtained could help design more suitable interventions to increase the awareness about alcohol environment and prevent hazardous and harmful alcohol consumption.
